# Microspatial variability in community structure and photophysiology of calcified macroalgal microbiomes revealed by coupling of hyperspectral and high-resolution fluorescence imaging

**DOI:** 10.1038/srep22343

**Published:** 2016-02-29

**Authors:** R. G. Perkins, C. J. Williamson, J. Brodie, L. Barillé, P. Launeau, J. Lavaud, M. L. Yallop, B. Jesus

**Affiliations:** 1 School of Earth and Ocean Sciences, Cardiff University, Cardiff, Glamorgan, CF10 3AT, UK; 2 Natural History Museum, Department of Life Sciences, Cromwell Road, London SW7 5BD, UK; 3 Université de Nantes, Labotoire de Mer Molécules Santé EA 2160, Faculté des Sciences et des Techniques, B.P. 92208, 44322 Nantes cedex 3, France; 4 LUNAM Université, Université de Nantes, Laboratoire de Planétologie et Géodynamique UMR 6112, Faculté des Sciences et des Techniques, B.P. 92208, 44322 Nantes cedex 3, France; 5 UMR7266 LIENSs ‘Littoral, Environnement et Sociétés’, CNRS/Université de La Rochelle, Institut du Littoral et de l’Environnement, La Rochelle, France; 6 School of Biological Sciences, Life Sciences Building, 24 Tyndall Avenue, Bristol, BS8 1TQ, UK; 7 University of Lisboa, Faculty of Sciences, BioISI – Biosystems & Integrative Sciences Institute, Campo Grande, 1749-016 Lisboa, Portugal

## Abstract

Calcifying coralline macroalgae provide biogenic habitats colonised by epiphytic microalgae that contribute significantly to community productivity. Georeferenced hyperspectral and high-resolution fluorescence imaging were coupled to microspatially mapped community composition and relative biomass of macroalgal host and epiphyte microalgal groups, and their weighted contributions to productivity within host fronds of *Corallina officinalis* on upper and lower zones of a rocky shore were determined. Lower shore epiphytes were dominated by filamentous diatoms (Bacillariophyta), confined to the apex of the frond structure, which were low light acclimated but retained a high capacity for photoprotective down regulation and contributed up to 51% of total community productivity. Upper shore epiphytes were dominated by green algae (Chlorophyta) and single-celled diatoms (principally *Cocconeis* spp.), which were high light acclimated but present at far lower relative biomass and contributed negligibly to productivity. The host, *C. officinalis* was the main primary producer. Variation in light environment resulting from differences in shore height and shading within the host macroalga, likely play a large role in determining patterns in epiphyte community structure, biomass and productivity observed. Additionally, microspatial gradients in photophysiological parameters along the host macroalga likely resulted from age-dependent variation in pigments as well as the gradient in light environment.

Epiphytes are well known to play important roles in enhancing productivity and biodiversity within marine macrophyte ecosystems, with extensive research on epiphyte community composition and ecosystem function for seagrass ecosystems^1^,^2^,^3^,^4^,^5^,^6^,^7^. However, epiphyte communities on macroalgae have received far less attention with few studies investigating epiphyte physiology or productivity. The dominance of diatoms within epiphyte communities of macrophytes is important as they account for 40% of coastal productivity, often exceeding the productivity of the host macrophyte^8^. Snoeijs^9^,^10^ reported that diatoms were the main epiphytes on the macroalgal hosts studied at sites in the Baltic Sea and that community structure varied temporally on a seasonal basis and along a salinity gradient. Al-Handal and Wulff^11^ showed that epiphytic diatoms of macroalgal hosts on the Antarctic Peninsula preferentially colonised firstly Rhodophyta then Phaeophyceae and finally Chlorophyta, with species such as *Cocconeis* spp., *Entopyla australis*, *Grammatophora arctica*, *Licmophora antarctica* and *Pseudogomphonema kamtschaticum* dominating. In contrast, Main and MacIntyre^12^ observed no host specificity shown by epiphytes in the Yaquina estuary, Oregon, USA, and Majewska *et al*.^13^ concluded that site environmental factors were more influential than host specificity in the Terra Nova Bay, Ross Sea, Antarctica. Karsten *et al*.^14^ investigated, *ex situ*, the light and temperature preferences of two epiphytic diatoms collected from the brown algal species *Chordaria flagelliformis*, showing low light acclimation (<15 μmol photons m^−2^ s^−1^ PAR) and highest growth rates between 2 and 14 °C. Da Silva Costa *et al*.^15^ found that epiphytic diatom biomass was greatest on sheltered sites, with biraphid taxa dominating. This was one of the few studies that investigated the variation in community structure within the thallus of the host, in this case the rhodophyte *Galaxaura rugosa* in north-east Brazil. The present study investigated spatial variation in the structure and functioning of the epiphyte community growing on the host rhodophyte, *Corallina officinalis*.

*C. officinalis* is a calcifying red macroalga found in intertidal and shallow subtidal rocky habitats, where it acts as an important ecosystem engineer, dominating climax communities^16^,^17^,^18^,^19^. *Corallina* species often form extensive macroalgal turfs that cover large areas of the intertidal and provide substratum, habitat and refugia for a number of important marine organisms^20^,^21^,^22^,^23^,^24^. *C. officinalis*, like other con-generics, is an articulated (geniculate) coralline alga, consisting of branched flexible fronds attached to crustose holdfasts^16^. The fronds are made up of small calcified segments (intergenicula), separated by un-calcified nodes (genicula)^16^. This morphology results in a heterogeneous complex substratum, which facilitates colonization by benthic microalgal taxa comprising a mixed community of diatoms, Chlorophyta and Cyanophyta^25^,^26^.

In this study, the spatial variation in photosynthetic microalgal epiphytes on the host *C. officinalis* was investigated on both a large scale (across shore heights on a rocky shore) and small scale (within individual fronds), with measurements made concomitantly for the epiphyte community and the host frond. This was achieved using a novel combination of state-of-the-art hyperspectral and high-resolution variable chlorophyll fluorescence imaging (see Ralph *et al*.^27^ for an overview of fluorescence imaging). This enabled pairing of productivity and photophysiological parameters with pigment data to generate a highly resolved spatial map of the *C. officinalis* biome. The hypotheses of the study were that, 1. *C. officinalis* provides a suitable substratum for microalgal epiphytes, with community structure varying spatially as a result of shore height and within host frond structure, 2. Plasticity of photophysiology and productivity of both the host and the epiphytes would be a function of light dose and intensity, e.g. with low light acclimation on the low shore and in shaded regions within the *C. officinalis* frond, and 3. Epiphyte productivity is a major contribution to the overall community productivity, dependent upon the dominant epiphyte taxa present.

## Results

### Epiphyte distribution across the rocky shore

**Figure 1:**
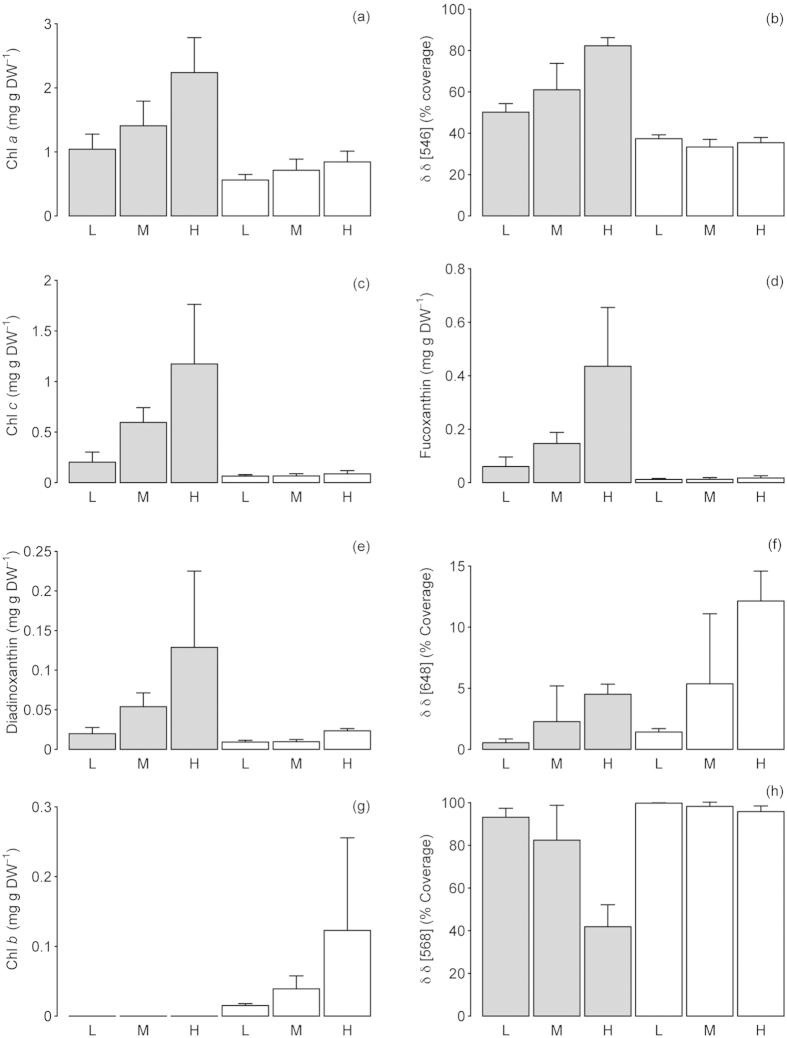
Pigments measured by chemical extraction and HPLC (**a,c,d,e,g**) and second derivative (δδ) hyperspectral imaging analysis **(b,f,h**) for chlorophyll a (**a**), fucoxanthin derivative at 546 nm as an indicator of diatom relative abundance (**b**), chlorophyll c (**c**), fucoxanthin (**d**), diadinoxanthin (**e**), chlorophyll b derivative 648 nm as an indicator of Chlorophyta relative abundance (**f**), chlorophyll b (**g**) and phycoerythrin derivative at 568 nm as an indicator of host *C. officinalis* relative abundance (**h**). Grey bars indicate lower shore samples and white bars refer to upper shore samples for epiphyte biomass categorised as low (L), medium (M) and high (**h**). Mean data with S.E bars, N = 4.

Total phototrophic biomass of the community (community biomass being the combined *C. officinalis*, epiphytic diatom and Chlorophyta) as indicated by extracted Chl a (Fig. 1a) showed a higher biomass on the lower shore compared to the upper shore, with incremental increases from low, to medium to high-epiphyte biomass category. On the lower shore the epiphyte community was dominated by diatoms with highly significant correlations between the hyperspectral measurement of relative abundance of diatoms (fucoxanthin, δδ 546 nm; Fig. 1b) and pigment data for Chl c (r = 0.96, p < 0.01; Fig. 1c), fucoxanthin (r = 0.98, p < 0.01; Fig. 1d) and diadinoxanthin (r = 0.95, p < 0.01; Fig. 1e). Indices of diatom abundance also indicated that diatoms were present on the upper shore, but at far lower biomass than on the lower shore. In comparison, epiphytic Chlorophyta were more abundant in the upper shore epiphyte community compared to the lower shore (Fig. 1f), with a significant correlation between the hyperspectral relative abundance of Chlorophyta (δδ 648 nm) and Chl b (r = 0.93, p < 0.01; Fig. 1g). However, hyperspectral data did show Chlorophyta to be present on the lower shore (Fig. 1f), whereas pigment data did not show the same pattern (Fig. 1g). Combined, the summed hyperspectral abundance for diatoms and Chlorophyta effectively dominated the pattern in Chl a, with a highly significant correlation (r = 0.995, p < 0.001). As a result of the epiphytic masking of the underlying *C. officinalis* hyperspectral relative abundance (phycoerythrin, δδ 568 nm; Fig. 1h), there was a highly significant negative correlation between phycoerythrin and Chl a on the lower shore (r = −0.97, p < 0.01).

**Figure 2:**
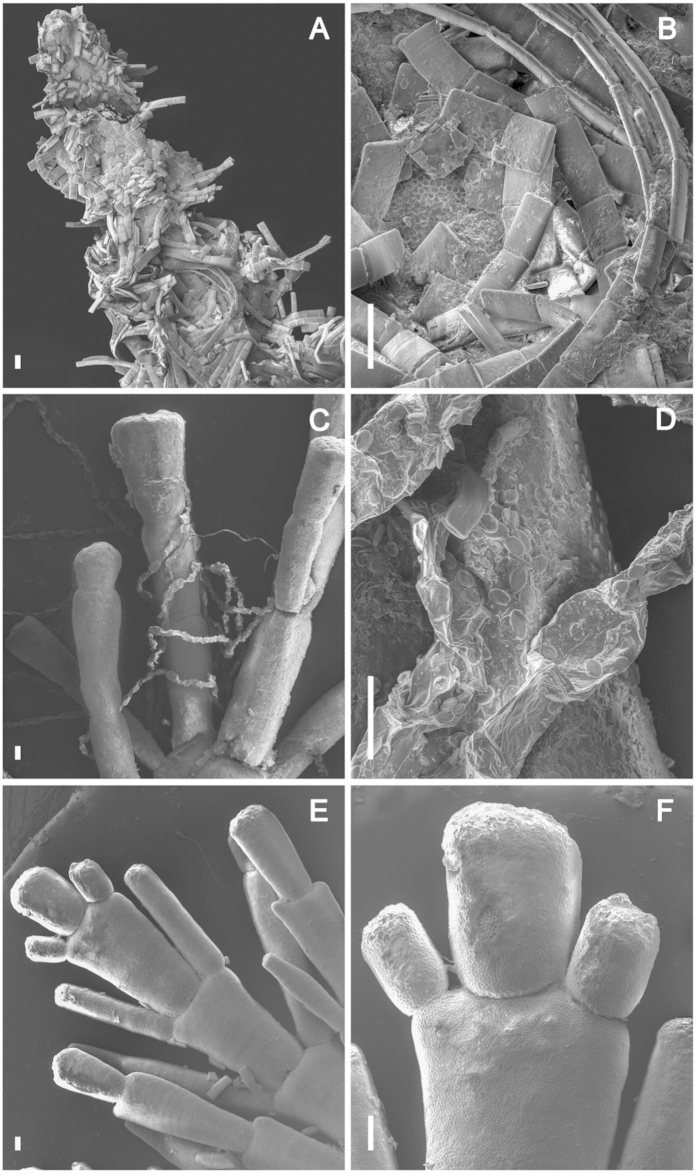
Scanning electron micrographs (scale bar indicates 10 μm) of *C. officinalis* fronds with epiphytes. (**A,B**) frond apical tip from lower shore with high-biomass of epiphytes, showing dominance of filamentous diatoms; (**C,D**) frond from upper shore with high-biomass of epiphytes, showing filamentous green algae and single-celled diatoms (mainly *Cocconeis* spp.); (**E,F**) frond apical tip from upper shore with low-biomass of epiphytes.

All pigments, except for Chl b, were significantly greater in high-epiphyte biomass, compared to medium biomass, which in turn was greater than low-epiphyte biomass, (Chl a, F_2,18_ = 8.12, p < 0.005; Chl c, F_2,18_ = 7.67, p < 0.01; fucoxanthin, F_2,18_ = 7.13, p < 0.01; diadinoxanthin, F_2,18_ = 23.51, p < 0.001; all post hoc analysis, Tukey’s test, at p < 0.05). Additionally, all pigments were significantly higher in concentration on the lower shore than on the upper shore (Chl a, F_1,18_ = 43.8, p < 0.001; Chl c, F F_1,18_ = 124.2, p < 0.001; fucoxanthin, F_1,18_ = 118.3, p < 0.001; diadinoxanthin, F_1,18_ = 70.94, p < 0.001) other than Chl b, which was only present in samples from the upper shore. SEM images of the lower shore fronds confirmed the dominance of diatoms (Fig. 2A,B) with filamentous species appearing most abundant, possibly masking single-celled species such as *Cocconeis* spp. (principally *C. scutellum*), which were more prevalent on the upper shore alongside epiphytic Chlorophyta (Fig. 2C,D). In contrast, images of low-epiphyte biomass fronds (Fig. 2E,F) showed an almost total absence of epiphytes, hence indicating “uncontaminated” hyperspectral and fluorescence images of *C. officinalis* fronds for pigment analysis and productivity (see below) measurements of the host macroalga.

### Epiphyte distribution within the host macroalga

**Figure 3:**
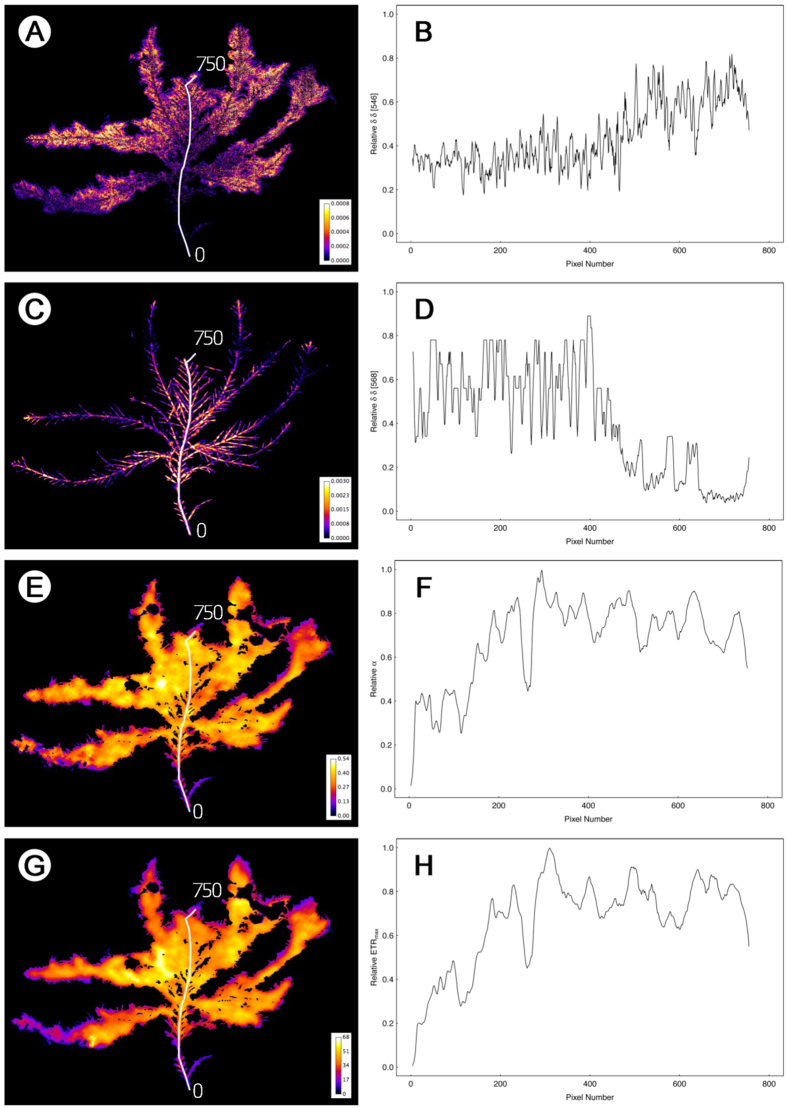
Hyperspectral images and associated transect derivative data for diatoms at 546 nm (**A,B**) and *C. officinalis* at 568 nm **(C,D**) and corresponding georeferenced fluorescence images and associated transects of light response curve parameters α (**E,F**) and ETR_max_ (**G,H**) for a low shore frond with high-biomass of epiphytes dominated by filamentous diatoms. The white lines on panels (**A,C,E,G**) demonstrate positioning of the transect used to produce the pixel thresholded data in panels (**B,D,F,H**). The transect length (0 to 750 pixels) is indicated on each image. Note the visible dominance of diatoms in panel A corresponding to the fluorescence imager data in panels (**E,G**), and the increase in ETR_max_ and α along the transect towards the apical tip. The width of the *C. officinalis* frond was 6 cm.

**Figure 4:**
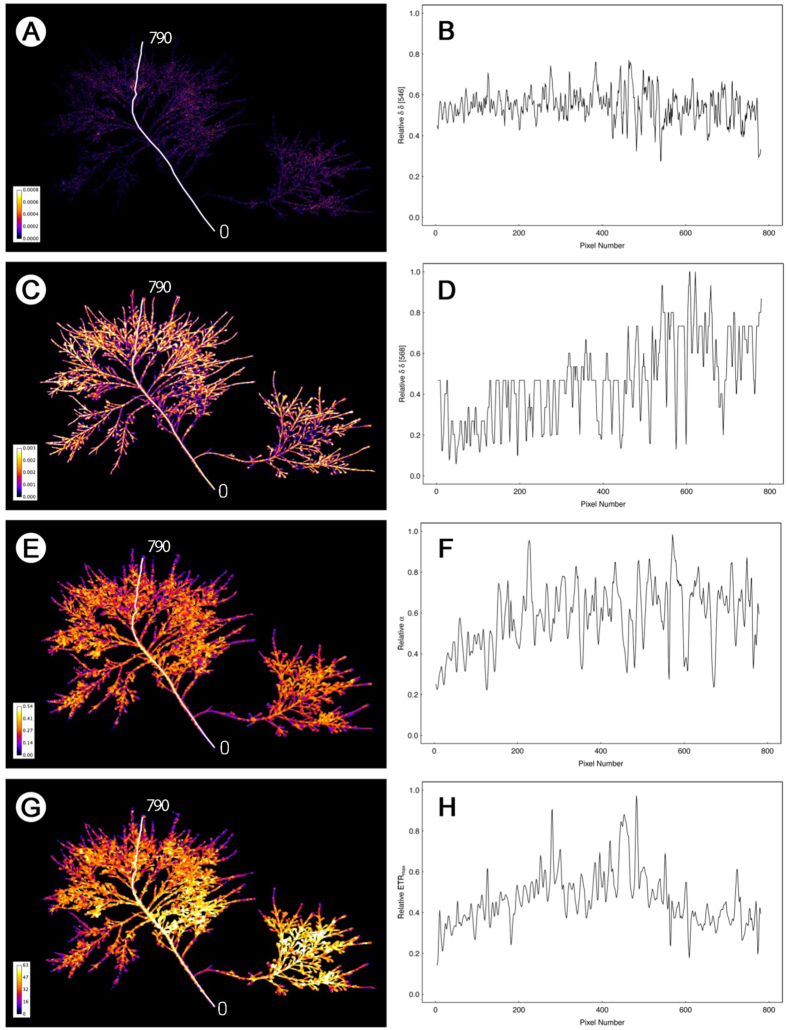
Hyperspectral images and associated transect derivative data for diatoms at 546 nm (**A,B**) and *C. officinalis* at 568 nm (**C,D**) and corresponding georeferenced fluorescence images (**E,G**) and associated transects of light response curve parameters α (**E,F**) and ETR_max_ (**G,H**) for an upper shore frond with low-biomass of epiphytes. The white lines on panels (**A,C,E**,**G**) demonstrate positioning of the transect used to produce the pixel thresholded data in panels (**B,D,F,H**). The transect length (0 to 750 pixels) is indicated on each image. Note the visible low abundance of epiphytic diatoms in panel A and the relatively stable ETR_max_ and α along the transect. The width of the *C. officinalis* frond was 6.5 cm.

Hyperspectral imaging enabled transect analysis along the length of each *C. officinalis* frond, with individual pixel resolution analysis of each wavelength corresponding to red algal phycoerythrin signal (δδ 568 nm), diatoms (δδ 546 nm) and Chlorophyta (δδ 648 nm). In total, four transects were carried out along four *C. officinalis* fronds from lower and upper shore at each epiphyte loading category (low/middle/high). An example of the data is shown in Fig. 3 for a high-epiphyte abundance lower shore frond and Fig. 4 for a low-epiphyte abundance upper shore frond. For the lower shore frond, false colour imaging (Fig. 3A) showed that diatoms were largely confined to the outer (towards the apex) two thirds of the frond with the δδ 546 nm signal (Fig. 3B) showing an almost linear increase in relative abundance of diatoms along the transect from base to apex of the frond. In contrast, the *C. officinalis* signal of phycoerythrin (δδ 568 nm, false colour image Fig. 3C) decreased significantly from 400 pixels outwards along the frond, corresponding to the point where diatom biomass increased. Hence the surface layers of epiphytic algae masked the subsurface red algal biomass signal. These patterns were evident for all lower shore fronds, across each epiphyte loading category. On the upper shore, high- and middle-epiphyte abundance fronds showed the same patterns, with the hyperspectral signals for diatoms (δδ 546 nm), and additionally Chlorophyta (δδ 648 nm), combining to increase towards the outer region of the frond, masking the subsurface *C. officinalis* signal for phycoerythrin. Given the far lower abundance of diatoms along the entire length of low-epiphyte biomass upper shore fronds (see example Fig. 4A–D), no masking of the red algal biomass signal was observed along frond transects (Fig. 4C,D), with host *Corallina* signal showing a linear increase in relative biomass from the basal region of the frond towards the apical tip.

Geo-rectifying of imaging-PAM to hyperspectral data then enabled the overlay of fluorescence images of photosynthetic rapid light curve parameters and construction of transects along frond axes. Along transects, the light utilisation coefficient (α, Fig. 3E) and the relative maximum electron transport rate (ETR_max_, false colour image Fig. 3G) both showed the same patterns, with increasing magnitude from 0 (frond base) to around 300 pixels, followed by variable but largely constant high values over the rest of the frond. Similar patterns were seen for all transects on both upper and lower shore medium- and high-epiphyte biomass fronds. Again, in contrast, the low-epiphyte biomass upper shore frond showed far less of a pattern in rapid light curve parameters α and ETR_max_ (Fig. 4E–H). α showed a slight increase over the first 200 pixels from the frond base (Fig. 4F) and ETR_max_ (Fig. 4H) showed a comparative increase mid-frond compared to both the basal and apical regions. These patterns were representative of all low-epiphyte biomass upper shore fronds. Data demonstrated the relative contributions of the epiphyte community to the overall measurements of ETR_max_ and α, and enabled the proportional pixel sub-setting of *C. officinalis*, diatoms and Chlorophyta to photophysiological measurements to be determined (see next section).

### Photophysiology of C. officinalis and epiphyte community components

**Figure 5:**
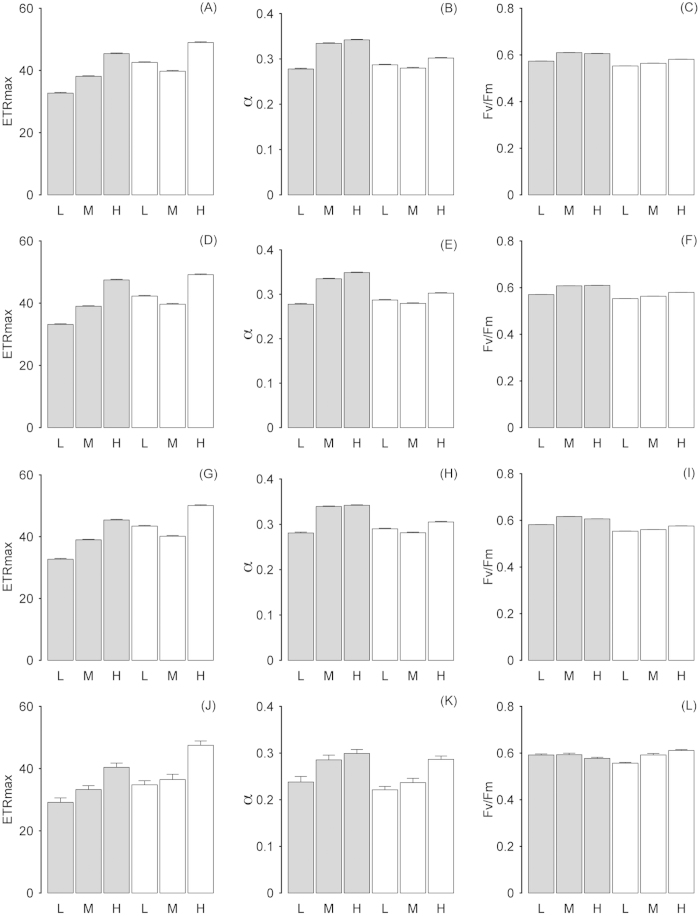
Rapid light curve parameters ETR_max_ (left hand column) and α (middle column) and maximum light use efficiency *F*_*v*_*/F*_*m*_ (right hand column) for the whole community dataset (**A–C**, thresholded as Chl a), *C. officinalis* (**D–F**, thresholded as phycoerythrin), diatoms (**G–I**, thresholded as fucoxanthin) and Chlorophyta (**J–L**, thresholded as Chl b). Data are shown for the lower shore (grey bars) and upper shore (white bars) for epiphyte biomass categorised as low (**L**), medium (M) and high (**H**). Mean data with error bars, N = 4.

Across the entire frond, rapid light curve (RLC) parameters followed the same trends for each component of the *C. officinalis* community (combined community, Fig. 5A–C; *C. officinalis* frond, Fig. 5D–F; diatoms, Fig. 5G–I; Chlorophyta, Fig. 5J–L). The relative electron transport rate (ETR_max_) showed a highly significant correlation with the light saturation coefficient (E_k_), showing the same patterns for each dataset and so E_k_ data are not shown. For the combined community, ETR_max_ increased significantly (F_2,120000_ = 4185, p < 0.001) as epiphyte biomass increased on lower shore samples and largely also on upper shore samples, and was significantly greater (F_1,120000_ = 2955, p < 0.001) for the upper shore compared to the low shore (Fig. 5A). However the low-epiphyte samples on the upper shore had slightly higher ETR_max_ than the medium-epiphyte biomass samples (significant at p < 0.05, post hoc Tukey test). The coefficient of light use efficiency (α) also increased concomitantly with ETR_max_ as epiphyte biomass increased (F_2,120000_ = 1494, p < 0.001), and was significantly higher (F_1,120000_ = 2259, p < 0.001) on the lower shore than on the upper shore (Fig. 5B). The dark adapted quantum efficiency (*F*_*v*_*/F*_*m*_; Fig. 5C) was significantly higher on the lower shore than on the upper shore (F_1,120000_ = 4797, p < 0.001) and also significantly increased (F_2,120000_ = 12777, p < 0.001) as epiphyte biomass increased. *C. officinalis* frond data for rapid light curve parameters showed the same patterns as for the community data with significantly higher ETR_max_ (F_1,120000_ = 1752, p < 0.001; Fig. 5D) on the upper shore, and significantly higher *F*_*v*_*/F*_*m*_ (F_1,120000_ = 13696, p < 0.001; Fig. 5C) and α (F_1,120000_ = 2700, p < 0.001; Fig. 5E) on the lower shore. *C. officinalis* frond ETR_max_ (F_2,120000_ = 5162, p < 0.001), α (F_2,120000_ = 1793, p < 0.001) and *F*_*v*_*/F*_*m*_ (F_2,120000_ = 5574, P < 0.001) all increased significantly as biomass increased. Epiphytic diatoms also showed the same patterns as for the community data with significantly higher ETR_max_ (F_1,120000_ = 3657, p < 0.001; Fig. 5G) on the upper shore, and significantly higher *F*_*v*_*/F*_*m*_ (F_1,120000_ = 20574, p < 0.001; Fig. 4I) and α (F_1,120000_ = 2310, p < 0.001; Fig. 5H) on the lower shore. Diatom ETR_max_ (F_2,120000_ = 4343, P < 0.001), α (F_2,120000_ = 1407, p < 0.001) and *F*_*v*_*/F*_*m*_ (F_2,120000_ = 3128, p < 0.001) all increased significantly as biomass increased. Epiphytic Chlorophyta showed similar patterns as for the community data with significantly higher ETR_max_ (F_1,120000_ = 97.2, p < 0.001; Fig. 5J) on the upper shore and significantly greater α (F_1,120000_ = 54.8, p < 0.001; Fig. 5K) on the lower shore. However there was no significant difference in *F*_*v*_*/F*_*m*_ between shore heights (Fig. 5L). Chlorophyta ETR_max_ (F_2,120000_ = 178.3, p < 0.001; Fig. 4), α (F_2,120000_ = 107.3, p < 0.001) and *F*_*v*_*/F*_*m*_ (F_2,120000_ = 42.1, p < 0.01) all increased significantly as biomass increased (although lower shore high-epiphyte biomass had a slightly lower *F*_*v*_*/F*_*m*_, but note that Chlorophyta were at very low biomass on the lower shore). There was a high significant interaction (p < 0.001) between factors frond and epiphyte biomass for all the above statistical tests for rapid light curve parameters, except for *F*_*v*_*/F*_*m*_ for Chlorophyta which was significant at p < 0.01.

**Figure 6:**
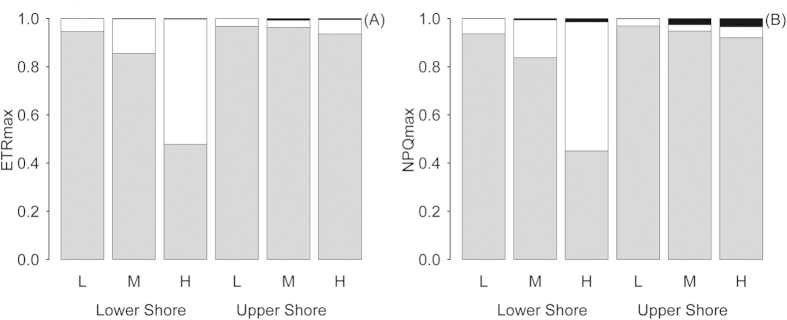
Weighted contributions of *C. officinalis* (grey), diatoms (white) and Chlorophyta (black) to ETR_max_ (**A**) and NPQ (**B**) as a proportion of the community total values for lower and upper shore and for epiphyte biomass categorised as low (L), medium (M) and high (H). Note the increasing contribution of diatoms to ETR_max_ and NPQ on the lower shore proportional to the increase in epiphyte biomass.

The relative contribution to the biome productivity (using ETR_max_ as a proxy) of the three dominant components of the *Corallina* and epiphyte community was calculated as the product of the weighted hyperspectral derivative and ETR_max_ for each epiphyte biomass category at both shore heights (Fig. 6A). The host *C. officinalis* contributed by far the most to the biome productivity (typically between 85 ± 7.1 and 96 ± 1.1%), except for the lower shore high-epiphyte biomass category, when diatoms increased to account for 51 ± 8.4%. Chlorophyta biomass was proportionally low and as a result contributed between 0 and 2.2 ± 0.41% of total ETR_max_, the maxima being for the medium biomass epiphyte category on the upper shore. It should be noted that diatoms contributed to productivity on the upper shore to a greater extent than the Chlorophyta, albeit at a far lower level than on the lower shore. Down regulation of photochemistry measured as non-photochemical quenching (NPQ, Fig. 6B) showed a near identical pattern to that of ETR_max_, with the *C. officinalis* signal dominating on the upper shore for all levels of epiphyte biomass and also on the lower shore, except for the high-epiphyte biomass category, when the measurement was dominated by diatoms.

## Discussion

This study has demonstrated the high contribution of epiphytic diatoms to community productivity within a *Corallina officinalis* biome, but only on the lower shore where diatom biomass was significantly higher than other shore regions. Findings suggest that light environment plays an important role in control of epiphyte biomass and species succession and/or zonation, such that single-celled, adnate diatoms and Chlorophyta colonise and dominate the upper shore, but were outcompeted on the lower shore by filamentous diatoms (although single-celled diatoms were also possibly present at significant biomass within the matrix of low shore epiphytes). Spatially, epiphyte biomass varied across the shore and also within the algal frond, with corresponding patterns in photosynthetic productivity (ETR_max_) and other photophysiological variables. The community productivity was largely dominated by the host macroalga, *C. officinalis*, except where low shore epiphyte biomass was greatest and dominated by diatoms (when productivity was largely equal between diatoms and the host). This is therefore in contrast to other studies which have emphasised the importance of epiphyte productivity, although these are primarily on seagrasses^1^,^2^,^3^,^4^,^5^,^6^,^7^, with fewer studies on macroalgae^8^.

Our data demonstrate a high sensitivity and reliability of hyperspectral imaging to detect pigments indicative of algal groups. Specifically, through the pairing of hyperspectral images with HPLC pigment analysis and SEM, we have shown the ability of hyperspectral imaging for effective mapping of macroalgal phototrophic microbiomes. There is evidence, for diatoms at least, that different types of macroalgal host thallus offer different microclimates and will influence epiphyte composition^28^. The present study focussed on *C. officinalis*, a geniculate species with calcified intergenicula interspersed with un-calcified genicula^29^, whose surface morphology is likely to preferentially select for certain types of epiphyte and indirectly influence productivity. Our data demonstrate greatest epiphyte biomass on the lower shore, with SEM imaging indicating dominance by filamentous diatoms. These may have masked other taxa, such as single-celled diatoms, Chlorophyta and Cyanophyta. Pigment data did not detect Chlorophyta on the lower shore, whereas hyperspectral imaging detected a comparatively low biomass of Chlorophyta, possibly indicating a higher sensitivity for the hyperspectral imaging, as samples were paired for analysis with pigments. Lower shore diatom dominance was indicated by the hyperspectral data at a wavelength of 546 nm, corresponding with peak absorption by fucoxanthin, along with pigment extraction data for chlorophyll c, fucoxanthin and the xanthophyll pigment, diadinoxanthin. The patterns in diadinoxanthin indicated a relatively stable content of this precursor to diatoxanthin, which is used in the xanthophyll cycle to down regulate photochemistry under increasing light level^30^. This would suggest a relatively stable level of capacity to induce non-photochemical quenching (NPQ, see below) by diatoms on the lower shore at all levels of epiphyte biomass. On the upper shore, diatoms were also present, but were largely single cells of *Cocconeis* species, alongside filamentous Chlorophyta (indicated by hyperspectral signal for chlorophyll b (δδ 648 nm) and pigment data), suggesting a spatial gradient in diatom taxa across shore heights.

Known gradients of stress across intertidal rocky shores explain the patterns in epiphyte abundance and community composition described above, i.e. reduced epiphyte abundance and community complexity higher up the shore. During tidal emersion periods, intertidal communities located on the upper shore experience a greater degree of abiotic stress due to the longer duration of emersion in comparison to the lower shore^31^,^32^,^33^. For example, Williamson *et al*.^33^ demonstrated that rock pool inhabiting *Corallina* experienced greater temperature and carbonate chemistry fluctuations, and significantly higher photo-dose, in upper-, as compared to lower-shore rock pools. Our data thus highlight an inverse relationship between abiotic stress and epiphyte abundance/community complexity of intertidal *Corallina* fronds. This is in agreement with the findings of Da Silva Costa^15^, who found that epiphytic diatom biomass was greatest in low stress, sheltered sites, and supports the assertion that environmental factors are more influential than host specificity in determining microbiome composition^13^. Additionally, the difference in community structure between the lower and upper shore may also represent a successional pattern from initial colonisers (*Cocconeis* spp. and Chlorophyta) on the upper shore to a more established biofilm of filamentous diatoms on the lower shore, as observed for freshwater biofilms^34^. Patterns in both epiphyte community composition and host/epiphyte photoacclimation reflected gradients in photodose across sampled shore heights. In addition to the overall lower epiphyte biomass observed on upper shore samples, a greater relative abundance of Chlorophyta, which are often more high light acclimated than diatoms^35^, was observed on the upper shore. On the lower shore, where emersion period is shorter and hence light dose lower^33^, it is likely that Chlorophyta are outcompeted by the filamentous colonial diatoms, which then form a more three-dimensional epiphyte community. This could afford an advantage through self-shading during periods of high ambient light level (e.g. a midday low tide), comparable to the self-shading reported for microalgae in fluorescence studies of mixed communities on the surface of stromatolites^36^. Photoacclimation enhances efficiency of light harvesting (i.e. high α) in low light environments and maximises productivity (i.e. high ETR_max_) in high light^7^,^37^. During the present study, low light acclimation on the lower shore and high light acclimation on the upper shore were observed at the community level as well as for each individual epiphyte group, and for the *C. officinalis* host, with higher ETR_max_ on the upper shore and higher α on the lower shore across all measurements. Additionally, maximum light use efficiency, *F*_*v*_*/F*_*m*_, was greater on the lower shore, correlating with the pattern for α. This would be expected, as under low light phototrophs maximise light use efficiency through an increase in reaction centre capacity as well as light harvesting efficiency with a relative increase in Chl a^38^. Note that such photoacclimation is a response to photodose rather than photo-intensity. The former would differ at the sampling site due to a 2 to 3 h difference in duration of exposure (depending on neap to spring tide respectively), whereas intensity of incident irradiance would obviously not differ if both shore heights were exposed. Thus photoacclimation to a lower light dose, but still necessitating photoprotection (e.g. non-photochemical down regulation, NPQ, see below) against high light intensity would be required on the lower shore.

For the first time, intra-frond patterns in epiphyte colonisation twinned with photophysiology measurements are presented for a macroalga and epiphyte biome. Both diatoms and Chlorophyta were observed to increase in biomass on the apical two thirds of *C. officinalis* fronds. This could be due to abrasion of the frond or, more likely, light limitation due to shading within the frond, as observed within seagrass blades^39^,^40^. Light acclimation was also observed along the frond, with ETR_max_ and α both increasing along transects from the base to the apical tip (in all samples other than low epiphyte biomass samples from the upper shore where ETR_max_ showed a mid-frond peak, decreasing slightly towards the apical tip), suggesting increasing photosynthetic capability overall as opposed to comparative high/low light acclimation^38^. These patterns are in close agreement with Ralph and Gademann^41^, who reported low ETR in the basal region, intermediate ETR in the middle and high ETR in the apical region of *Posidonia australis* seagrass blades due to photoacclimation to the prevailing light environment. The mid-frond peak in ETR_max_ for upper shore fronds with low epiphyte biomass, may have been due to photoinhibition induced by high light stress in the apical region of the *C. officinalis* fronds, such that ETR was down regulated. It is of note that increases in rapid light curve parameters occurred from pixel 0 (base) to 300 during the present study, whereas epiphyte biomass was observed to increase from pixel 400 outwards along the frond transect. This suggests an increasing photosynthetic capacity of the host *C. officinalis* from base to tip, which was then largely masked by the epiphyte signal on the outer apical half of the frond. However, pixel thresholding enabled sub-setting of the relative contributions of the host and each component of the epiphyte community and hence would partly remove this masking effect (see later discussion of productivity). Therefore data almost certainly indicate light limitation towards the basal region of *C. officinalis* fronds, limiting both the host productivity and the ability of epiphytes to colonise. Additionally, increases in host ETR_max_ and α with distance from the frond base may be related to an ageing process, with the basal region being the older part of the host frond^27^. Mazzella and Alberte^42^ reported a 3-fold increase in maximum photosynthetic rate along blades of the seagrass *Thalassia testudinum*, attributed to a gradient in down regulation and photodamage, whereas an age-dependent loss in *F*_*v*_*/F*_*m*_ was observed by Enriquez *et al*.^40^. Thus age-dependent factors along with light acclimation likely combine to account for the patterns in ETR_max_ and α observed for host *C. officinalis* fronds on both the upper and lower shore.

Productivity of the *C. officinalis* biome was clearly dominated by the host macroalga itself with regard to relative ETR_max_ proxy measurements. Thus the overall ETR_max_ signal was dominated by the phycoerythrin-weighted signal as a proportion of the total community measurement of ETR_max_. This would be due to the greater proportional biomass of the host macroalga and the absence of epiphytes closer to the basal region of the frond. Masking of the surface of *C. officinalis* would not have directly altered the detected measurements of ETR_max_ and other RLC parameters, as these were calculated for random pixels above a threshold for phycoerythrin signal (see Methods). However, it may be that the contribution of *C. officinalis* was over-estimated as overlying diatoms may have shaded the host frond, reducing the light level it was exposed to during the RLC. Given that photosynthetic quantum efficiency shows a negative curvilinear response to increasing light level^37^ and ETR_max_ is the product of this efficiency and the light level, a falsely high light level (due to shading) will result in a falsely high efficiency and hence an over estimated ETR_max_. This is directly comparable to shading effects in microalgal biofilms, which result in over estimation of ETR and ETR_max_^37^,^43^, and would increase the importance of the epiphytic diatoms to the community productivity. It should be noted that the interaction between epiphyte biomass and host photophysiology is likely to suppress host productivity through a shading effect, although findings showed *C. officinalis* to be the main contributor to productivity with respect to ETR_max_. The lower shore high biomass of epiphyte diatoms would likely shade the host frond and hence induce low light acclimation (lower ETR_max_ and higher α) and a higher pigment concentration for light harvesting as observed in this study. Thus the greater potential productivity indicated by higher ETR_max_ towards the apical region of *C. officinalis* fronds was due to the contribution of the epiphytic diatoms rather than the host frond.

Diatoms on lower shore fronds were shown to significantly contribute to community productivity, especially for the high-epiphyte biomass category of samples. ETR_max_ and α also both increased significantly as epiphyte biomass increased, indicating greater photosynthetic capability rather than simple low/high photoacclimation. Concomitantly, down regulation capability through NPQ induction increased, suggesting that the high productivity and high NPQ induction typical of diatoms^44^,^45^ were competitive advantages for the colonial filamentous diatoms on the lower shore. Merino *et al*.^46^ demonstrated spatial patterns in NPQ within *Thalassia testudinum* seagrass blades as a result of the prevailing light gradient, similar to those observed in this study. It is suggested here that the capability for diatoms to induce high levels of photoprotective down regulation is partly responsible for their dominance as macroalgal epiphytes, coupled with their high photic plasticity shown by their ability to acclimate to relatively lower light on the lower shore and within an algal frond.

In conclusion, this study has shown that *C. officinalis* provides a suitable substratum for a range of epiphytes, notably diatoms and green algae. The epiphytic microalgal community within the biogenic *C. officinalis* biome varied in community composition and contribution to productivity as a function of both the surface morphology of the host and environmental parameters, probably dominated by light, in agreement with studies of seagrasses^27^,^40^,^41^,^42^,^46^, but also temperature and other stresses that vary between the upper and lower intertidal^33^,^47^. Lower shore epiphytes were dominated by diatoms that were capable of contributing up to 51% of community productivity. In contrast, upper shore epiphyte biomass was far lower and consisted of a mixed community of Chlorophyta and diatoms, which contributed to less than 10% of the community productivity. Within the macroalgal frond, epiphytes were restricted to half to two thirds of the apical frond surface, probably due to light limitation closer to basal regions. Productivity increased towards the outer tip of the frond, correlating with an increase in epiphyte biomass and also host pigment density (possibly an age-dependent variable and/or low light acclimation), indicating microspatial photoacclimation, productivity and biomass variability, as well as spatial patterns across the rocky shore overall. The relative importance of epiphyte productivity within a macroalgal biome is therefore partly a function of light (likely both dose and intensity) within the algal frond and as a function of shore height. Findings have important implications in our understanding of the balance between host and epiphyte communities and the associated ecosystem services of the community, which (for the better studied seagrass meadows) is associated with genetic and species diversity^48^.

## Methods

### Sampling and sample site

Samples of *C. officinalis* (whole fronds including the basal holdfast) were collected from tidal pools at La Pointe St-Gildas located in the French Atlantic coast at two shore heights, i.e. lower shore (47° 8′11.75“N, 2°14′57.95“W) and upper shore (47° 8′9.29“N 2°14′48.27“W). Twenty fronds were collected at each shore height, selecting a wide variety of specimens from different holdfasts and with different levels of epiphyte coverage, ranging from fronds that were visually (by naked eye, later verified by microscopy) free of epiphytes to specimens with dense epiphyte coverage obscuring the host frond.

### Hyperspectral imaging and derivative analysis

Hyperspectral images were acquired using a HySpex VNIR 1600 camera that produces 160 images in the VIS-NIR spectrum (400 to 1000 nm) with a spectral resolution of 4.5 nm. Samples were illuminated by a halogen light and placed at a distance of 50 cm from the camera at a position resulting in images with square pixels and a spatial resolution of 0.2 mm. Pixel reflectance values were obtained by dividing each pixel by the mean intensity of a Spectralon® plate (~99% reflectance). Reflectance images were further processed into second derivative images because second derivative values can amplify minute changes in the reflectance spectra, allowing the separation of pigment absorption features from the different taxonomic groups^49^. Second derivative images (δδ) were calculated following Jesus *et al*.^50^ and second derivative peaks were used to assign pigments to absorption wavelengths. Namely, second derivative values at 546 nm (δδ 546 nm) were used to produce fucoxanthin images (diatoms), second derivative values at 568 nm (δδ 568 nm) were used to produce phycoerythrin images (*C. officinalis*), second derivative values at 648 nm (δδ 648 nm) to produce chlorophyll *b* images (Chlorophyta), and second derivative values at 677 nm (δδ 677 nm) were used to produce chlorophyll *a* images (whole community)^50^.

### Fluorescence imaging

All pulse amplitude modulated (PAM) fluorescence measurements were carried out using an Imaging PAM (Walz, Maxi version) with a blue measuring light. Imaging methods largely followed those of Ralph *et al*.^27^. Rapid light photosynthesis-irradiance curves (RLCs)^37^,^51^ were constructed using 10 incremental light steps (1, 11, 21, 36, 56, 111, 186, 281, 336, 531 μmol photons m^−2^ s^−1^) of 30 s each. RLCs were carried out using ImagingWin software (Walz, ImagingWin v2.33) and data were exported as multi-layered Tiff files. These files were first imported into ImageJ^52^ using a custom made macro function that imports the Tiff files as “stack” images and calculates PSII quantum efficiency images for each step, i.e. *F*_*v*_/*F*_*m*_ for the first light step (dark) and *F*_*q*_’/*F*_*m*_’ for the remaining light steps. Finally, PSII quantum efficiency images for each light step were saved as individual text files (matrices) before being imported in R v.3.0.2^53^ where they were converted in the equivalent electron transport rate (ETR) matrix values using: ETR = *F*_*q*_’/*F*_*m*_’ × 0.5 × PAR. For each pixel a light photosynthesis-irradiance curve model was fitted using the model of Platt *et al*.^54^ and new images were produced for each model parameter, i.e. α (light utilisation coefficient), E_k_ (light saturation coefficient) and ETR_max_ (maximum ETR). Concomitantly, down regulation in the form of non-photochemical quenching (NPQ) was calculated from the change in maximum fluorescence yield^35^,^44^,^45^ (*F*_*m*_ – *F*_*m*_’)/*F*_*m*_’. Iterative curve fitting to NPQ as a function of light curve irradiance then permitted the calculation of the maximum level of NPQ^37^ induced during the RLC (NPQ_max_), hence comparable in methodology to the determination of ETR_max_.

### Data analysis and GIS

To enable direct comparison of hyperspectral and imaging-PAM data, imaging-PAM images were geo-rectified to hyperspectral images using the georeference GDAL plugin of QGIS (v.2.6.1. ‘Brighton’). Per sample, a points coordinates layer was constructed by selecting conspicuous locations (e.g. branch tips) on the infra-red imaging-PAM output loaded into the GDAL plugin, and visually matching these to the same locations on the reference hyperspectral RGB image loaded into the QGIS map canvas. A minimum of 20 reference points were selected per sample. Points layers were then used to geo-rectify imaging-PAM images using a polynomial 3 linear transformation. The quality of transformation was assessed based on the mean transformation error (<1 in all cases) and by visual comparison of geo-rectified images against reference RGB hyperspectral images. Given the higher resolution of hyperspectral images, geo-rectified imaging-PAM images were re-resolved in QGIS by cropping to the extent of the hyperspectral images and matching the horizontal and vertical layer resolution.

In order to extract data values from those pixels only corresponding to *C. officinalis* frond and/or epiphyte, a polygon layer covering the extent of these pixels was manually prepared per sample, by tracing the extent of frond/epiphytes on RGB hyperspectral images in QGIS. Hyperspectral and processed imaging-PAM images were then clipped with their corresponding polygon layer in R (v.3.0.2.) using the mask function of the raster package^55^. Manual construction of polygon layers was preferred to e.g. selecting pixels based on RGB values, given difficulties encountered with the simultaneous recovery of different epiphyte groups. Following the clipping of images, pixel data for each hyperspectral and imaging-PAM layer were extracted per sample using the getValues function of the raster package. Pixels with ‘NA’ values recorded for any photophysiological parameter were excluded from subsequent analyses.

Separation of pixel data into respective *C. officinalis*/epiphyte groups, i.e. host, diatom or Chlorophyta pixels, was achieved by sub-setting pixels based on threshold second derivative values; *C. officinalis* = δδ 568 nm > 0; diatoms = δδ 546 nm > 0; Chlorophyta = δδ 648 nm > −0.00025. Threshold values were determined based on visual assessment of pixel recovery per group. Where single pixels demonstrated second derivative values greater than thresholds for multiple groups (568 nm, 546 nm, 648 nm), these pixels (and associated photophysiological data) were affiliated with multiple groups in subsequent analyses. The relative abundance (%) of each algal group per sample (*C. officinalis*, diatoms and Chlorophyta) was calculated as the number of pixels recovered per group (based on the above thresholds), normalised to the total number of pixels of the entire ‘community’ (Chla, δδ 677 nm) per sample.

To investigate the existence of axial gradients on *C. officinalis* fronds, transects were drawn over each image (reflectance and RLC fluorescence parameters) using the “Segmented line” tool in ImageJ (1.47v) and pixel values extracted using the tool “Measure” in the same software. Four replicate transects were repeated per frond at each shore height and within each epiphyte biomass category. Data were then processed as above to determine variation of epiphyte biomass and photophysiological parameters as a function of position within the frond.

### Scanning Electron Microscopy

*C. officinalis* samples were freeze-dried over 48 h and gold coated before observation in a JEOL 7600F scanning electron microscope. For each treatment, *C. officinalis* samples were cut in three equal parts following the frond main axis in order to analyse the spatial heterogeneity from the base to the tip of the frond. The examples presented here are representative of the most common observations of epiphyte groups on fronds of high-epiphyte biomass and fronds with visually low-epiphyte biomass. Thus images corroborated visual selection of epiphyte biomass category and also enabled partial identification of dominant epiphyte taxa.

### Pigment analysis

Entire *C. officinalis* fronds were frozen in liquid nitrogen and stored at −80 °C until pigment extraction. A sub-sample of freeze-dried frond (between 140 and 400 mg) was ground and pigments extracted during 15 min at −20 °C with 15 ml of 95% cold buffered methanol (2% ammonium acetate). Samples were sonicated for 30 s, centrifuged and filtered (Whatman membrane filters, 0.2 μm) immediately before injecting 100 μL in the HPLC (Dionex, Ultimate 3000 RS). Pigments were separated using a C18 reverse phase chromatographic column preceded by a pre-column. The elution solvents were 1M of ammonium acetate in methanol (80:20) and methanol-acetone (60:40) following the same elution gradient as described in Méléder *et al*.^56^ (2005). Identification and calibration of the HPLC peaks was confirmed with chlorophyll a (Chl a), chlorophyll b (Chl b), β-carotene, chlorophyll c (Chl c), fucoxanthin (fuco), diadinoxanthin (DD) and diatoxanthin (DT) standards from DHI Laboratory Products. All pigments were expressed by dry weight, i.e. mg g^−1^.

### Statistical analysis

All statistical analyses and plotting of data were performed using R v.3.0.2 (R Core Team 2014) and ImageJ (1.47v). Prior to all analyses, normality of data was checked by visual inspection of frequency histograms and qq-plots constructed using the car package^57^. With the exception of pigment data, given the large sample sizes involved in statistical analyses (e.g. up to 120,000 data points), formal tests of normality and homogeneity of variance were not suitable. Statistical assumptions were thus checked by examination of model criticism plots following the application of models to data as described below. For pigment data, normality and homogeneity of variance were tested prior to analyses using the Shapiro-Wilk test and Levene’s test, respectively. If assumptions were not met, Box-Cox power transformation was applied using the boxcox function of the MASS package^58^ and assumptions re-checked.

Differences in the derivative-based relative abundance of algal groups (*C. officinalis*, diatoms, Chlorophyta) between shore-heights (lower/upper) and epiphyte loading categories (low/medium/high), were examined separately per algal group, using 2-way Analysis of Variance (ANOVA), with the fixed factors ‘shore height’ (2 levels) and ‘epiphyte loading’ (3 levels), with interaction. Differences in photophysiological parameters (α, E_k_, ETR_max_ and *F*_*v*_/*F*_*m*_)^53^ between shore-heights and epiphyte loading categories were examined separately per derivative-based algal group (whole ‘community’, *C. officinalis*, diatom, or Chlorophyta pixels). Given the differing number of pixels recovered per group across samples, a random subset of each group’s pixels was selected for analysis from each sample, allowing a balanced ANOVA approach. For whole ‘community’, *C. officinalis* and diatom derivative-based groups, n = 5000 pixels were randomly selected from each sample for analyses, whilst n = 500 Chlorophyta pixels were randomly selected from Chlorophyta pixels of each sample given the reduced Chlorophyta abundance across samples. Differences in photophysiological parameters were then analysed using a nested-ANOVA design, with the fixed factor ‘shore height’ (2 levels), and the factor ‘sample’ (12 levels) nested within ‘epiphyte loading’ (3 levels). Differences in pigments (Chl a, Chl b, Chl c, fucoxanthin and diadinoxanthin) between shore-heights and epiphyte loading categories were analysed with 2-way ANOVA as described above. Post-hoc Tukey honest significant differences analysis was used to examine significant differences highlighted by all ANOVA tests. Correlation analyses between variables were carried out using Pearson product-moment correlation in PAST statistical software.

## Additional Information

**How to cite this article**: Perkins, R. G. *et al*. Microspatial variability in community structure and photophysiology of calcified macroalgal microbiomes revealed by coupling of hyperspectral and high-resolution fluorescence imaging. *Sci. Rep*. **6**, 22343; doi: 10.1038/srep22343 (2016).
